# Associations between COVID-19 Vaccination Status and Self-Reported SARS-CoV-2 Infection among 8538 Children Aged 3–17 Years during a Massive COVID-19 Outbreak after China Changed Its Zero-COVID-19 Policy: A Cross-Sectional Survey

**DOI:** 10.3390/vaccines11091401

**Published:** 2023-08-22

**Authors:** Lixian Su, Siyu Chen, Hongbiao Chen, Yuan Fang, Weijun Peng, Xiaofeng Zhou, Jingwei Luo, Xue Liang, Kechun Zhang, Zixin Wang

**Affiliations:** 1 Department of Child Healthcare, Shenzhen Futian District Maternity & Child Healthcare Hospital, Shenzhen 518016, China; xiandansumei@163.com; 2 Centre for Health Behaviours Research, JC School of Public Health and Primary Care, Faculty of Medicine, The Chinese University of Hong Kong, Hong Kong, China; chensiyu@link.cuhk.edu.hk (S.C.); 1155187741@link.cuhk.edu.hk (X.L.); 3 Longhua District Centre for Disease Control and Prevention, Shenzhen 518109, China; gesila2021@163.com (H.C.); igeneral@126.com (W.P.); zhouxiaofeng5184@163.com (X.Z.); mei257758@163.com (J.L.); zkc1317@yeah.net (K.Z.); 4 Department of Health and Physical Education, The Education University of Hong Kong, Hong Kong, China; fyuan@eduhk.hk

**Keywords:** COVID-19 vaccination, SARS-CoV-2 infection, children, living with COVID-19, China

## Abstract

This study aimed to investigate the associations between COVID-19 vaccination status and self-reported SARS-CoV-2 infection among children and adolescents aged 3–17 years during a massive COVID-19 outbreak after China changed its zero COVID policy. A cross-sectional online survey was conducted between 1 and 9 March 2023. Participants were the parents of children studying in kindergartens, primary schools, or secondary schools in Shenzhen. Convenient sampling was used. All kindergartens, primary schools, and secondary schools in the Longhua District of Shenzhen invited the parents of children and adolescents attending the schools. Interested parents completed an online survey. Multivariate logistic regression was fitted. Among 8538 participants, 40.9% self-reported that their children had SARS-CoV-2 infection after 7 December 2022, where 92.9% of them received two doses of the COVID-19 vaccines, and 74.6% received their second dose for more than six months. In multivariate analysis, children who received their second dose of the COVID-19 vaccination for no more than three months had a lower SARS-CoV-2 infection rate compared to unvaccinated children (<1 month: AOR: 0.17, 95% CI: 0.07, 0.44; 1–3 months: AOR: 0.54, 95% CI: 0.41, 0.75). The duration of protection conferred by the primary COVID-19 vaccination series was relatively short among children. A booster dose should be considered for children.

## 1. Introduction

China has implemented a zero-COVID policy since December 2019. On 7 December 2022, the country relaxed its strict COVID-19 control measures [1]. Centralized quarantine for people with SARS-CoV-2 infection, close contacts and cross-border travelers, universal regular COVID-19 nucleic acid amplification testing (NAAT), territory lockdown, and border control were discontinued [2]. People in China no longer needed a valid negative COVID-19 testing result before entering public spaces or taking public transportation [2]. Such dramatic changes in COVID-19 control measures marked the transition from a zero-COVID policy to “living with COVID”.

Soon after the changes in policy, a massive COVID-19 outbreak mainly caused by the Omicron variant of SARS-CoV-2 hit China. Mathematic models estimated that 75% of Chinese people were infected with SARS-CoV-2 within two weeks after the policy change [3,4]. The infection rate within one month after the policy change was as high as 87.5–92.3% [3,4]. However, the actual COVID-19 situation was largely unknown during this wave of outbreak. The country stopped tracking and recording asymptomatic SARS-CoV-2 cases since 14 December 2022 and discontinued updating daily COVID-19 situation since 25 December 2022 [5]. Moreover, since the capacity of NAAT could not meet the dramatically increasing demands during the outbreak, many people in China could not access COVID-19 testing in time [2]. Under such conditions, a large-scale population-based survey became an alternative way to understand the COVID-19 situation in China.

COVID-19 seriously affected the health of children and adolescents [6,7]. Previous studies showed that children and adolescents under the age of 18 years accounted for 17.5% of all SARS-CoV-2 cases in the United States [8] and 16.7% in Germany [9]. This study focused on preschool children and school-age children and adolescents aged 3–17 years, as they were more vulnerable to COVID-19 due to the frequent teacher-student interactions and class/group activities at campus [10]. The COVID-19 vaccination is important to protect children and adolescents against SARS-CoV-2 infection and its severe consequences. Studies consistently showed that children and adolescents receiving at least two doses of the mRNA COVID-19 vaccine (BNT162b2) had a lower risk of infection and hospitalization caused by the Omicron variant of SARS-CoV-2, as compared to those who were unvaccinated [11]. Among fully vaccinated children aged 5–11 years, the vaccine effectiveness against any symptomatic or asymptomatic Omicron infection 14–82 days after receipt of the second dose of BNT162b2 was 31% [12]. Such vaccine effectiveness among children and adolescents aged 12–15 years was 59% [12]. There was fewer evidence about the vaccine effectiveness of the inactivated COVID-19 vaccines used by children in China. One study estimated that the vaccine effectiveness of CoronaVac was 38.2% against symptomatic COVID-19 caused by the Omicron variant among children aged 3–5 years in China [13]. Another study indicated that the CoronaVac had similar efficacy in preventing hospitalization as the BNT162b2 vaccine among children aged 5–11 years [14].

China commenced the administration of COVID-19 vaccines to adolescents between the ages of 12 and 17 in July 2021, and extended the initiative to encompass children aged 3 to 11 in October 2021 [15]. Children and adolescents are recommended to receive inactivated COVID-19 vaccines [15]. The country reached a high coverage of COVID-19 vaccination among children and adolescents within a short time [6]. According to the official report, over 90% of children and adolescents aged 3–17 years in the country completed the primary COVID-19 vaccination series by April 2022 [16]. However, a booster dose of COVID-19 vaccine is not yet available for children or adolescents under the age of 18 years. Hence, there are concerns about the waning protection of the primary vaccination series among children and adolescents in China.

To address the information and service gap, a large-scale online survey was conducted among the parents of children and adolescents aged 3–17 years in Shenzhen, China. This study aimed to investigate the association between COVID-19 vaccination status and self-reported SARS-CoV-2 infection among preschool children and school-age children and adolescents aged 3–17 years. We hypothesized that children and adolescents who had received two doses of the COVID-19 vaccines would have a lower likelihood of SARS-CoV-2 infection compared to those who were unvaccinated.

## 2. Materials and Methods

### 2.1. Study Design

A cross-sectional online survey was conducted among the parents of children aged 3–17 years in Shenzhen, China, between 1 and 9 March 2023.

### 2.2. Participants and Data Collection

Participants of this online survey were: (1) aged 18 years or above, (2) had at least one child who was attending kindergartens, primary schools, or secondary schools in Shenzhen at the time of the survey, and (3) had access to a smartphone with internet access. This study was conducted in the Longhua District of Shenzhen. This study was implemented by the Longhua District Center for Disease Control and Prevention (CDC). All 220 kindergartens and 105 primary and secondary schools in the Longhua District of Shenzhen participated in this study. All these schools have established WeChat groups involving the teachers and parents of all children and adolescents attending the schools for delivering school notices. Convenient sampling was performed in this study, where the staff of the participating schools sent an invitation letter that included an information sheet about this study through the WeChat groups. The invitation letter explained that participation is voluntary, refusals had no consequences, and the study will not gather personal contact information or identification. The data will remain confidential and be solely used for research purposes. Interested parents could access the online questionnaire by scanning a quick response (QR) code in the invitation letter. Online informed consent was obtained before the participants started to answer the questionnaire.

Three commercial online survey platforms are most widely used in China. They are Sojump, Diaoyanbao, and Questionnaire Star. Their features are similar to their western counterparts. All these survey platforms provide: (1) templates to speed the design of questionnaire, (2) compatibility to link with social media, (3) options to distribute incentives, and (4) support to recruit participants [17]. The collected data can be exported in common data formats [17]. All these platforms are shown to be reliable [17]. Among these platforms, Questionnaire Star is the most popular one because it has a free version and allows distribution of virtual cash incentives through WeChat [17]. In this study, we used Questionnaire Star (Changsha Ranxing Information Technology Co., Changsha, China) to develop the online questionnaire. Each WeChat account was permitted to access the online questionnaire only once to prevent duplicate responses. The survey consisted of a total of 38 items, with an average of 15 items per page across 3 pages. On average, it took the participants approximately 10 min to complete the survey. The survey platform conducted a completeness check before each questionnaire submission. Participants were given the opportunity to review and modify their responses using a “back” button. If there were multiple children under the age of 18 in their household, participants were instructed to refer to the child whose birthday was closest to the survey date when answering questions [6]. No incentive was given to the participants. All data were stored in the online survey platform server and protected by a password. Only the first authors and the corresponding author had access to the database. A total of 8538 participants completed the survey between 1 and 9 March 2023, which accounted for about 10.7% of the total number of students in kindergartens, primary schools, and secondary schools in the Longhua District of Shenzhen at the beginning of 2023 (about 80,000). Ethics approval was obtained from the Longhua District CDC (reference number: 2021006).

### 2.3. Sample Size Planning

There are 80,000 preschool children and school-age children and adolescents attending kindergartens, primary schools, and secondary schools in the Longhua District at the time of this study. This study invited the parents of all these children and adolescents to complete the survey, and we expected 10% of these parents (*n* = 8000) would join the study. With a statistical power of 0.80 and an alpha value of 0.05, assuming that the SARS-CoV-2 infection rate in the reference group (with a protective factor) ranges from 10 to 30%, the sample size would be able to detect the smallest odds ratio (OR) of 1.14 between individuals with and without the protective factor (PASS 11.0, NCSS LLC, Kaysville, UT, the United States).

### 2.4. Measurements

#### 2.4.1. Questionnaire Development

A panel comprising of epidemiologists, clinicians, and CDC employees was established to create the questionnaire utilized in the present investigation. The questionnaire was pilot tested among 10 parents to assess its clarity and readability. The participants involved in the pilot study believed that the questions presented were readily comprehensible, while also finding the length of the questionnaire to be reasonable. The study did not involve the participation of these 10 parents. The panel made revisions and finalized the questionnaire based on the feedback provided by these parents.

#### 2.4.2. Background Characteristics of the Children and Adolescents

Participants reported the age and gender of their index child and the number of household members living with the index child. In addition, they were asked if the index child had some chronic condition and whether the child was performing physical activity regularly.

#### 2.4.3. Children and Adolescents’ History of SARS-CoV-2 Infection

Participants were asked whether the index child had SARS-CoV-2 infection confirmed via NAAT and/or rapid antigen testing (RAT). For those who self-reported a history of SARS-CoV-2 infection, some details were collected, including the date of diagnosis and the presence of COVID-19-related symptoms. COVID-19-related symptoms were assessed using a validated checklist [18].

#### 2.4.4. Children and Adolescents’ COVID-19 Vaccination Status

Participants were asked about the number of doses of COVID-19 vaccination received by the index child. Date of the most recent dose of vaccination was also collected.

### 2.5. Statistical Analysis

Descriptive statistics were presented. Differences in SARS-CoV-2 infection rates, presence of COVID-19-related symptoms, and COVID-19 vaccination history across different age groups of children were compared using Chi-square tests. China changed its COVID-19 control policy on 7 December 2022. Therefore, the dependent variable was the self-reported SARS-CoV-2 infection on or after 7 December 2022. An initial assessment was conducted using a univariate logistic regression model to analyze the relationship between each independent variable of interest (COVID-19 vaccination history, confirmed SARS-CoV-2 before 7 December 2022, and background characteristics). All variables with *p* < 0.05 in the univariate analysis were considered as candidates for a multivariate logistic regression model. Sub-group analysis for children aged 3–6 years, 7–12 years, and adolescents aged 13–17 years were conducted. In China, these age groups represented children and adolescents attending kindergartens (3–6 years), primary schools (7–12 years), and secondary schools (13–17 years). The study calculated crude odds ratios (OR), adjusted OR (AOR), and their corresponding 95% confidence intervals. The data analysis was performed using SPSS Statistics for Windows version 26.0 (IBM Corp, Armonk, NY, USA). A significance level of *p* < 0.05 was considered statistically significant.

## 3. Results

### 3.1. Background Characteristics of the Participants

Over half of the children and adolescents were male (55.6%) and aged 7–12 years (54.6%). The majority of the children were living with at least three other household members (81.7%) without any chronic conditions (98.9%) and performing physical activity regularly in the past six months (73.2%) (Table 1).

### 3.2. History of SARS-CoV-2 Infection

**Figure 1:**
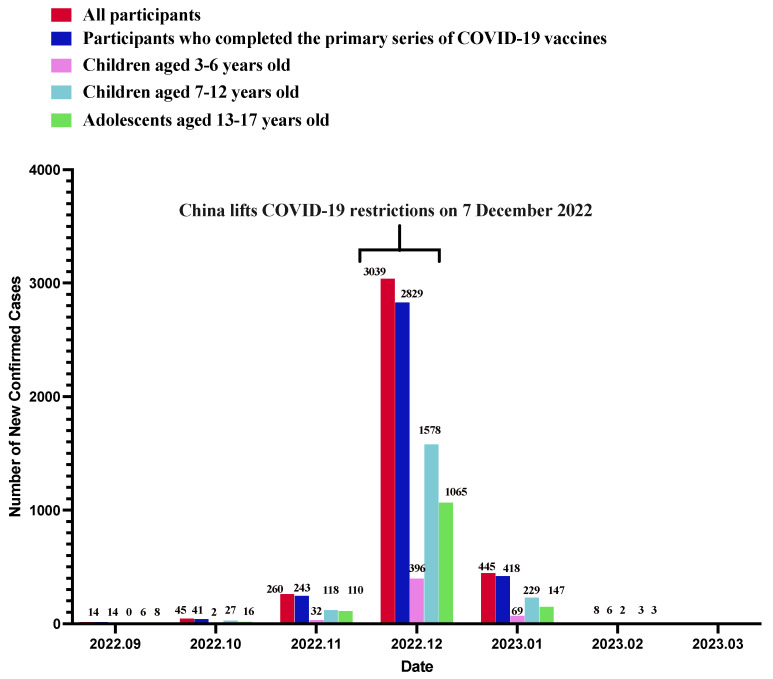
The number of new confirmed cases among the participants.

**Figure 2:**
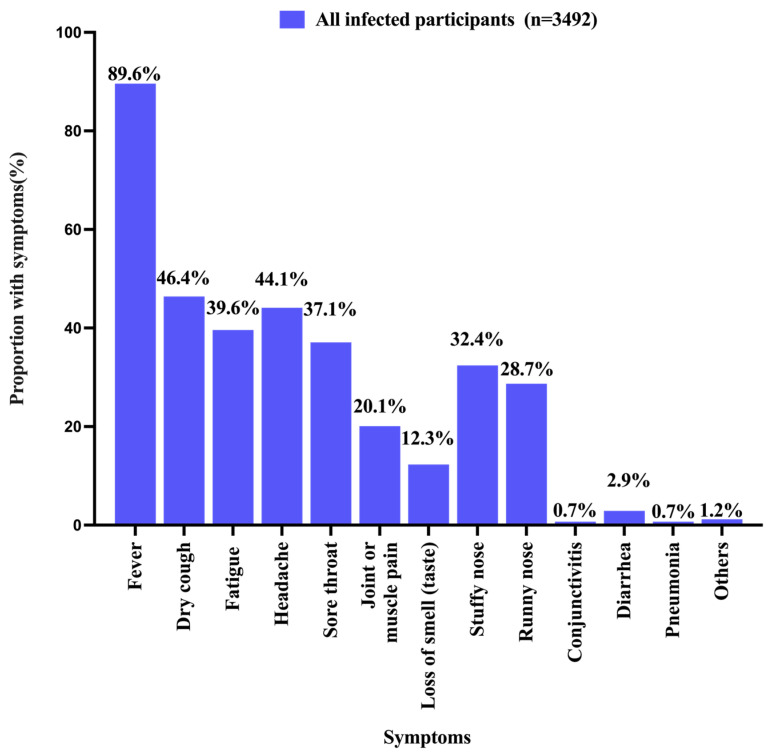
The proportion of COVID-19-related symptoms among all participants.

**Figure 3:**
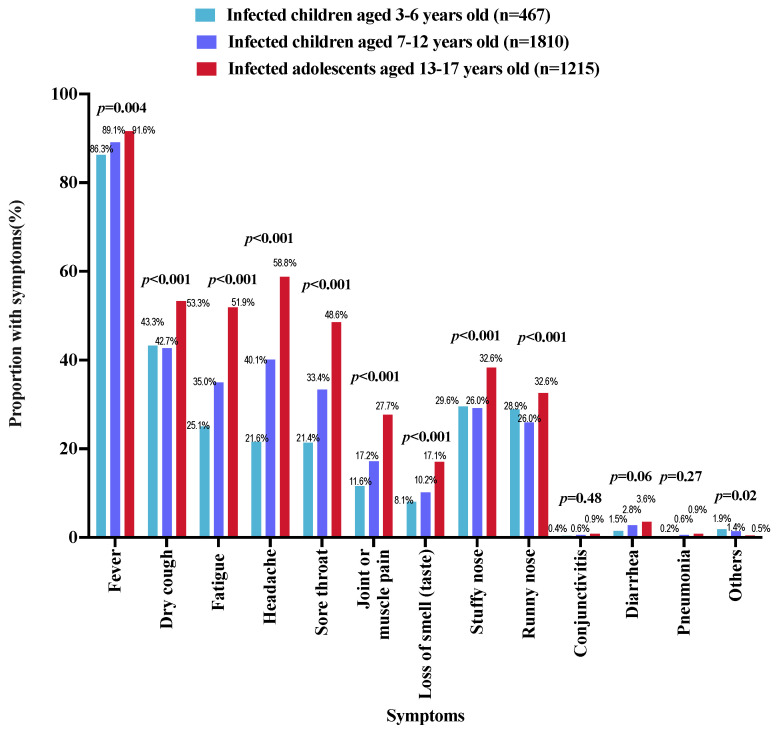
The proportion of COVID-19-related symptoms among participants with different ages.

**Figure 4:**
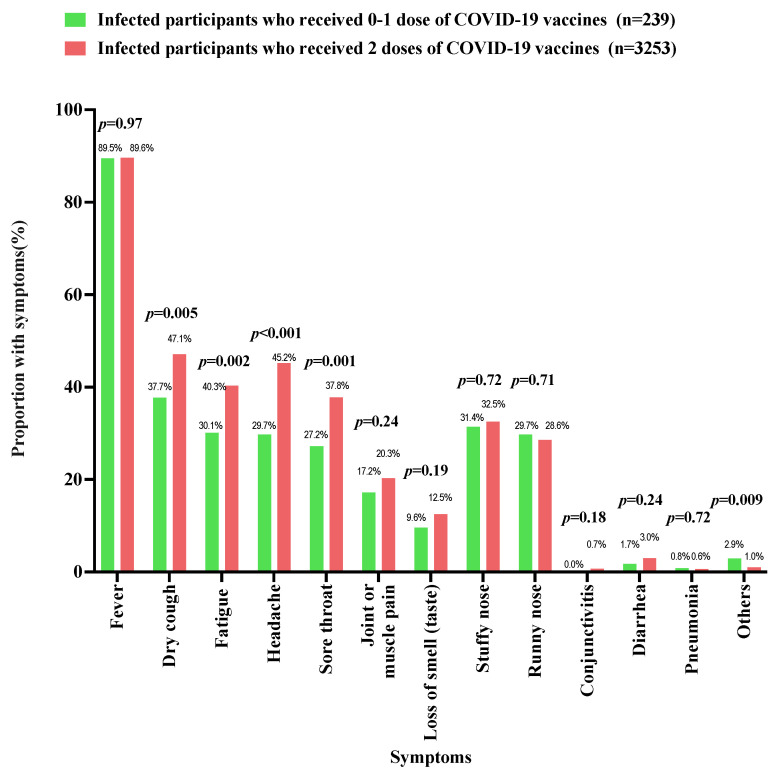
The proportion of COVID-19-related symptoms among participants with different COVID-19 vaccination statuses.

As shown in Table 1, 40.9% (*n* = 3492) of the parents self-reported that their index children had SARS-CoV-2 infection confirmed via NAAT (*n* = 1386, 39.7%), RAT (*n* = 1900, 54.4%), or both methods (*n* = 206, 5.9%) on or after 7 December 2022. Children and adolescents of a younger age had a lower SARS-CoV-2 infection rate as compared to their older counterparts (3–6 years: 35.9%; 7–12 years: 38.8%; 13–17 years: 47.3%; *p* < 0.001). The peak of infection was observed in December 2022 (Figure 1). All children and adolescents with SARS-CoV-2 infection on or after 7 December 2022 had at least one COVID-19-related symptom. The most common symptoms were fever (3129/3492, 89.6%), followed by dry cough (1621/3492, 46.4%), headache (1540/3492, 44.1%), and fatigue (1382/3492, 39.6%) (Figure 2). As compared to the younger children, adolescents aged 13–17 years with SARS-CoV-2 infection were more likely to have fever, dry cough, fatigue, headache, sore throat, joint or muscle pain, loss of smell, stuffy nose, and running nose (Figure 3). Compared to children and adolescents with SARS-CoV-2 infection who had received two doses of the COVID-19 vaccines, those who received 0–1 dose showed fewer symptoms such as dry cough, fatigue, headache, and sore throat (Figure 4).

Very few participants self-reported that their index children had SARS-CoV-2 infection before 7 December 2022 (4.4%). The majority of these infections occurred in November 2022 (260/379, 68.6%). None of these 379 children or adolescents reported confirmed SARS-CoV-2 infection on or after 7 December 2022.

### 3.3. History of COVID-19 Vaccination

Most children and adolescents received two doses of the COVID-19 vaccines (92.9%), and all vaccinated children received inactivated COVID-19 vaccines. The completion rate of the primary vaccination series (two doses) was significantly higher among adolescents aged 13–17 years compared to children aged 3–6 years (96.6% versus 77.7%, *p* < 0.001) or those aged 7–12 years (96.6% versus 95.2%, *p* = 0.007). About three quarters of the participants (6101/8180, 74.6%) reported that there was more than six months between their children’s second dose of COVID-19 vaccines before the changes in COVID-19 policy (7 December 2022). The proportion of children who had received their second dose for more than six months was higher among those aged 7–12 years (71.3%) and 13–17 years (72.7%), as compared to those aged 3–6 years (58.4%) (Table 1).

### 3.4. Association between COVID-19 Vaccination Status and SARS-CoV-2 Infection among All Participants

In multivariate analysis, children who received their second dose of the COVID-19 vaccination for no more than three months before 7 December 2022 had a lower likelihood of SARS-CoV-2 infection compared to unvaccinated children (<1 month: AOR: 0.17, 95% CI: 0.07, 0.44; 1–3 months: AOR: 0.54, 95% CI: 0.41, 0.75). Compared to unvaccinated children, those who received their second dose for more than 12 months was associated with a higher likelihood of SARS-CoV-2 infection (AOR: 1.33, 95% CI: 1.05, 1.67) (Table 2).

### 3.5. Association between COVID-19 Vaccination Status and SARS-CoV-2 Infection among Different Sub-Groups of Participants

Among children who received two doses of the COVID-19 vaccination, the longer interval between their second dose and December was associated with higher infection rates (Supplementary Tables S1 and S2).

Sub-group analysis among children and adolescents of different age groups showed different results regarding the association between the status of the COVID-19 vaccination and SARS-CoV-2 infection. Children between the ages of 3 and 6 who had received their second COVID-19 vaccination dose for no more than six months had a lower likelihood of SARS-CoV-2 infection compared to unvaccinated children of the same age group (1–3 months: AOR: 0.53, 95% CI: 0.29, 0.90; 4–6 months: AOR: 0.60, 95% CI: 0.38, 0.95). Among children aged 13–17 years, only those who received their second dose for less than one month had lower infection rates compared to unvaccinated participants (AOR: 0.11, 95% CI: 0.01, 0.88). The COVID-19 vaccination did not demonstrate an association with a reduced incidence of SARS-CoV-2 infection rates among children aged 7–12 years (Table 2).

## 4. Discussion

This study provided some useful first-hand data about the COVID-19 situation among children and adolescents in China after the country changed its COVID-19 policy. The findings also contributed evidence about the association between vaccination with inactivated COVID-19 vaccines and symptomatic SARS-CoV-2 among children and adolescents during the Omicron predominance. Since no official data about children was released during this period, the findings may have implications on policymaking and vaccination program planning for children and adolescents in China and other countries that mainly used inactivated COVID-19 vaccines.

In this study, 40.9% of the parents self-reported their children had confirmed SARS-CoV-2 infection on or after 7 December 2022. The infection rate was likely to be underestimated as this study did not include children or adolescents with asymptomatic SARS-CoV-2 infection. Previous studies showed that approximately 47.2–92.7% of children had symptoms following Omicron infection [19,20]. Since universal and regular COVID-19 screening was discontinued and people lacked access to NAAT and RAT after the policy changes [2], asymptomatic children or adolescents were unlikely to receive SARS-CoV-2 testing. Moreover, some children with COVID-19-related symptoms might not be able to receive SARS-CoV-2 testing in time due to the limited access to NAAT and RAT [2]. The self-reported SARS-CoV-2 infection rate within this single wave of outbreak was similar to the cumulative infection rate among children in England (48.2%) [21] and was much higher than those reported in Italy (8.1%) [22] and Brazil (3.6%) [22] in 2022.

Some reasons might explain the high infection rate among children and adolescents in China during this wave of outbreak. On the one hand, the COVID-19 situation was well-controlled in China before the policy changes and very few children or adolescents with a history of SARS-CoV-2 infection were reported before December 2022 (4.4% in the study). Therefore, most children lacked protection conferred by natural SARS-CoV-2 infection. A previous study showed that protection conferred by natural SARS-CoV-2 infection was strong and persistent among children [23]. In this study, none of the children or adolescents with SARS-CoV-2 infection before December 2022 had confirmed infection again between December 2022 and March 2023. On the other hand, the protection conferred by the COVID-19 vaccination may also be inadequate among children and adolescents in China. Among children and adolescents in the study, 92.9% received two doses of the COVID-19 vaccination before the policy changes, which was comparable to the official data released by the government in October 2022 [24]. The COVID-19 vaccination coverage among children and adolescents in China was comparable to those reported in the United Kingdom (98–99%) [25] and was slightly higher than those reported in the United States (77.6%) [26]. However, most Chinese children and adolescents received two doses of the COVID-19 vaccination soon after the national childhood vaccination was implemented in 2021 [16]. In this study, over 70% of the children received their second dose of the COVID-19 vaccination for more than six months. Similar to previous studies [13,14], the protection conferred by two doses of the inactivated vaccines against Omicron was modest. Receiving the second dose within 3 months was associated with a lower SARS-CoV-2 infection rate compared to unvaccinated children. However, the infection rate among those who had received the second dose for more than three months was not different from those who were unvaccinated. These findings suggested the waning effectiveness of inactivated COVID-19 vaccines among children and adolescents. A similar decrease in protection over time was observed among children who had received the mRNA vaccine (BNT162b2) [11]. Previous studies showed that receiving a third dose of BNA162b2 as a booster could increase vaccine effectiveness against symptomatic SARS-CoV-2 infection without safety concerns [11]. Regular monitoring children and adolescents’ COVID-19 situation and their immunogenicity to SARS-CoV-2 variants of concerns is needed to inform the implementation of the COVID-19 vaccine booster dose for children and adolescents in China. Many countries/regions, such as the United States and Hong Kong Special Administration Region of China, had been implementing a COVID-19 booster dose for children and adolescents aged 3 years or above [27,28].

The results also suggested that the association between the COVID-19 vaccination and self-reported symptomatic SARS-CoV-2 infection was different among children and adolescents of different age groups. In this study, children aged 3– 6 years who had received their second dose within 6 months had a lower SARS-CoV-2 infection rate. However, the SARS-CoV-2 infection rate was similar between children aged >6 years who had received their second dose for more than one month and unvaccinated children of the same age. Previous studies also observed different vaccine effectiveness of the BNT162b2 between children aged 3–4 years and those aged 5 years or above during the Omicron predominant [11]. Future studies should look at whether there are differences in immunogenicity to inactivated COVID-19 vaccines between younger and older children.

This study has several limitations. First, this was a cross-sectional and observational study, which could not establish causality. Second, it was a major limitation that our response rate was only 10.7%, which was lower than previous studies of similar topics in China [6,7,29,30]. As compared to these studies [6,7,29,30], our study did not involve face-to-face contact with the participants or incentives. In the literature, online surveys usually have a relatively low response rate [31]. The use of convenient sampling, low response rate, and not being able to collect information from refusals would lead to selection bias. The findings might not represent children and adolescents in Shenzhen. Third, the participants’ SARS-CoV-2 infection was self-reported by the parents without validation. Due to the concerns about stigma related to COVID-19 [32,33], parents might underreport their children’s SARS-CoV-2 infection due to social desirability. In addition, the self-reported data had recall bias. These biases reduced the accuracy of the reported infection rate. Fourth, the children’s COVID-19 vaccination status was also self-reported by the parents without validation. Parents might over report their children’s vaccination uptake due to social desirability. Some details of the vaccination (e.g., date) provided by the parents might be inaccurate due to recall bias. These issues might lead to bias when estimating the protective effects of COVID-19 vaccination. Fifth, the number of unvaccinated children was small in this study. Significant selection bias existed, which would affect the estimation of the association between COVID-19 vaccination status and SARS-CoV-2 infection. Sixth, since there were no asymptomatic SARS-CoV-2 cases in this study, this study only reflected the association between the COVID-19 vaccination and symptomatic SARS-CoV-2 infection. Moreover, the participants were only recruited from one Chinese city. Cautions should be taken when generalizing the findings to other Chinese cities. Shenzhen is one of the largest and most developed Chinese cities. Shenzhen had higher COVID-19 vaccination coverage and better supply of NAAT and RAT during the outbreak, as compared to less-developed Chinese cities. Furthermore, the utilization of other personal COVID-19 preventive measures (e.g., face mask wearing, hand washing, avoiding crowded places) among children during the outbreak was not measured. These measures are still useful in preventing Omicron [34]. The protective effects of COVID-19 vaccination might be overestimated.

## 5. Conclusions

In conclusion, receiving the second dose of the inactivated vaccine for no more than 3 months was associated with a lower likelihood of SARS-CoV-2 infection among children and adolescents compared to those who were unvaccinated. The duration of protection against Omicron conferred by the two doses of inactivated vaccines among children and adolescents was relatively short. Regular monitoring the children and adolescents’ COVID-19 situation and their immunogenicity to SARS-CoV-2 variants of concerns is needed to inform the implementation of the COVID-19 vaccine booster dose for children and adolescents in China.

## Figures and Tables

**Table 1:** Characteristics of the participants.

	All Participants (*n* = 8538)	Participants Aged 3–6 Years Old (*n* = 1302)	Participants Aged 7–12 Years Old (*n* = 4666)	Participants Aged 13–17 Years Old (*n* = 2570)	*p* Values
	*n* (%)	*n* (%)	*n* (%)	*n* (%)	
**Background characteristics**					
Sex assigned at birth					
Male	4748 (55.6)	713 (54.8)	2585 (55.4)	1450 (56.4)	0.56
Female	3790 (44.4)	589 (45.2)	2081 (44.6)	1120 (43.6)	
Number of other household members					
1	203 (2.4)	19 (1.5)	98 (2.1)	86 (3.4)	<0.001
2	1361 (15.9)	185 (14.2)	723 (15.5)	453 (17.6)	
3–5	6220 (72.9)	992 (76.2)	3431 (73.5)	1797 (69.9)	
>5	754 (8.8)	106 (8.1)	414 (8.9)	234 (9.1)	
Presence of any chronic conditions					
No	8444 (98.9)	1285 (98.7)	4621 (99.0)	2538 (98.8)	0.41
Yes	94 (1.1)	17 (1.3)	45 (1.0)	32 (1.2)	
Perform physical activity regularly (≥3 days/week of physical activity of at least 30 min/day in the past six months)					
No	2284 (26.8)	470 (36.1)	1239 (26.6)	575 (22.4)	<0.001
Yes	6254 (73.2)	832 (63.9)	3427 (73.4)	1995 (77.6)	
**History of SARS-CoV-2 infection**					
Self-reported SARS-CoV-2 infection on or after 7 December 2022					
No	5046 (59.1)	835 (64.1)	2856 (61.2)	1355 (52.7)	<0.001
Yes	3492 (40.9)	467 (35.9)	1810 (38.8)	1215 (47.3)	
Self-reported SARS-CoV-2 infection before 7 December 2022					
No	8159 (95.6)	1263 (97.0)	4485 (96.1)	2411 (93.8)	<0.001
Yes	379 (4.4)	39 (3.0)	181 (3.9)	159 (6.2)	
**COVID-19 vaccination status**					
Number of doses of COVID-19 vaccine received by the children (interval between the second dose of COVID-19 vaccine and 7 December 2022)					
0 dose	358 (4.2)	184 (14.1)	124 (2.7)	50 (1.9)	<0.001
1 dose	246 (2.9)	107 (8.2)	100 (2.1)	39 (1.5)	
2 doses (<1 month)	51 (0.6)	8 (0.6)	28 (0.6)	15 (0.6)	
2 doses (1–3 months)	436 (5.1)	76 (5.8)	260 (5.6)	100 (3.9)	
2 doses (4–6 months)	1493 (17.5)	168 (12.9)	827 (17.7)	498 (19.4)	
2 doses (7–9 months)	1486 (17.4)	195 (15.0)	800 (17.1)	491 (19.1)	
2 doses (10–12 months)	1040 (12.2)	191 (14.7)	589 (12.6)	260 (10.1)	
2 doses (>12 months)	3428 (40.1)	373 (28.7)	1938 (41.6)	1117 (43.5)	

N.A.: not applicable. *p* values were obtained using Chi-square tests comparing the difference in study variables between children of different age groups.

**Table 2:** Factors associated with self-reported SARS-CoV-2 infection on or after 7 December 2022 among the participants.

	All Participants (*n* = 8538)		Participants Aged 3–6 Years Old (*n* = 1302)		Participants Aged 7–12 Years Old (*n* = 4666)		Participants Aged 13–17 Years Old (*n* = 2570)	
	AOR (95% CI)	*p* Values	AOR (95% CI)	*p* Values	AOR (95% CI)	*p* Values	AOR (95% CI)	*p* Values
**Background characteristics**								
Age group, years								
3–6	Reference							
7–12	1.14 (1.00, 1.31)	0.049						
13–17	1.63 (1.41, 1.89)	<0.001	N.A.	N.A.	N.A.	N.A.	N.A.	N.A.
Sex assigned at birth								
Male	---		---		Reference		---	
Female	---	---	---	---	0.86 (0.76, 0.97)	0.01	---	---
Number of other household members								
1	Reference		Reference		---		Reference	
2	1.53 (1.12, 2.10)	0.008	4.29 (0.95, 19.25)	0.06	---	---	1.65 (1.02, 2.67)	0.04
3–5	1.51 (1.12, 2.04)	0.007	4.66 (1.06, 20.38)	0.04	---	---	1.47 (0.94, 2.31)	0.09
>5	1.33 (0.95, 1.85)	0.09	4.84 (1.06, 22.22)	0.04	---	---	1.09 (0.65, 1.83)	0.74
Presence of any chronic conditions								
No	---		---		---		---	
Yes	---	---	---	---	---	---	---	---
Perform physical activity regularly (≥3 days/week of physical activity of at least 30 min/day in the past six months)								
No	Reference		Reference		Reference		---	---
Yes	0.80 (0.73, 0.88)	<0.001	0.79 (0.62, 0.99)	0.047	0.79 (0.69, 0.90)	<0.001	---	---
Self-reported SARS-CoV-2 infection before 7 December 2022								
No	---		---		---	---	---	---
Yes	---	---	---	---	---	---	---	---
**COVID-19 vaccination status**								
Number of doses of COVID-19 vaccine received by the children (interval between the second dose of COVID-19 vaccine and 7 December 2022)								
0 dose	Reference		Reference		Reference		Reference	
1 dose	1.19 (0.86, 1.67)	0.30	1.26 (0.78, 2.04)	0.35	1.09 (0.63, 1.89)	0.77	1.36 (0.58, 3.19)	0.47
2 doses (>12 months)	1.33 (1.05, 1.67)	0.02	0.86 (0.60, 1.24)	0.42	1.59 (1.08, 2.33)	0.02	1.73 (0.97, 3.08)	0.07
2 doses (10–12 months)	0.98 (0.76, 1.26)	0.85	0.92 (0.60, 1.40)	0.69	1.10 (0.73, 1.66)	0.64	1.24 (0.67, 2.31)	0.49
2 doses (7–9 months)	1.02 (0.80, 1.30)	0.86	0.97 (0.64, 1.48)	0.90	1.14 (0.77, 1.70)	0.52	1.30 (0.72, 2.36)	0.39
2 doses (4–6 months)	0.80 (0.63, 1.02)	0.07	0.60 (0.38, 0.95)	0.03	1.04 (0.70, 1.55)	0.84	0.88 (0.48, 1.59)	0.67
2 doses (1–3 months)	0.54 (0.41, 0.75)	<0.001	0.53 (0.29, 0.90)	0.04	0.71 (0.45, 1.13)	0.15	0.50 (0.25, 1.04)	0.06
2 doses (<1 month)	0.17 (0.07, 0.44)	<0.001	N.A.	N.A.	0.33 (0.11, 1.02)	0.05	0.11 (0.01, 0.88)	0.04

N.A.: not applicable; ---: the variable with a *p*-value more than 0.05 in the univariate analysis was not included in the multivariate logistic regression models; AOR: the adjusted odds and odds ratios were derived from multivariate logistic regression models, incorporating all significant factors identified in the univariate analysis; CI: confidence interval.

## Data Availability

The datasets generated and/or analyzed during the current study are not publicly available as they contain sensitive personal behaviors but are available from the corresponding author on reasonable request.

## Supplementary Materials

The following supporting information can be downloaded at: https://www.mdpi.com/article/10.3390/vaccines11091401/s1, Table S1. Characteristics of the participants who had completed the primary series of COVID-19 vaccines (*n* = 7934). Table S2. Factors associated with COVID-19 infection after 7 December 2022, among the participants who completed the primary series of COVID-19 vaccines (*n* = 7934).
