# Supplementary material for: Associations between COVID-19 Vaccination Status and Self-Reported SARS-CoV-2 Infection among 8538 Children Aged 3–17 Years during a Massive COVID-19 Outbreak after China Changed Its Zero-COVID-19 Policy: A Cross-Sectional Survey
Source: Vaccines (Basel). 2023 Aug 22;11(9):1401. doi: 10.3390/vaccines11091401 (PMC10535453; doi:10.3390/vaccines11091401)
Supplement: Supplementary file 1 [file vaccines-11-01401-s001.zip › vaccines-2543866-supplementary.pdf]

Table S1. Characteristics of the participants who had completed the primary series of COVID-19 vaccines (n=7934)

	N	%
**Socio-demographic characteristics**		
Age group, years		
3-6	1011	12.7
7-12	4442	56.0
13-17	2481	31.3
Sex assigned at birth		
Male	4405	55.5
Female	3529	44.5
Number of other household members		
1	183	2.3
2	1255	15.8
3-5	5791	73.0
>5	705	8.9
Presence of chronic condition		
No	7855	99.0
Yes	79	1.0
Perform physical activity regularly (≥3 days/week of physical activity of at least 30 minutes/day in the past six months)		
No	2062	26.0
Yes	5872	74.0
**History of SARS-CoV-2 infection**		
Self-reported SARS-CoV-2 infection on or after 7 December 7 2022		
No	4681	59.0
Yes	3253	41.0
Self-reported SARS-CoV-2 infection before 7 December 2022		
No	7578	95.5
Yes	356	4.5
**COVID-19 vaccination status**		
Interval between the most recent dose of COVID-19 vaccine and 7 December 2022, month		
2 doses (<1 month prior to 7 Dec 2022)	51	0.6
2 doses (1-3 months prior to 7 Dec 2022)	436	5.5
2 doses (4-6 months prior to 7 Dec 2022)	1493	18.8
2 doses (7-9 months prior to 7 Dec 2022)	1486	18.7
2 doses (10-12 months prior to 7 Dec 2022)	1040	13.1
2 doses (>12 months prior to 7 Dec 2022)	3428	43.3

Table S2 Factors associated with COVID-19 infection after 7 December 2022 among the participants who completed the primary series of COVID-19 vaccines (n=7934)

	OR (95%CI)	*p* values	AOR (95%CI)	*p* values
**Socio-demographic characteristics**				
Age group, years				
3-6	Reference		Reference	
7-12	1.23 (1.07, 1.42)	0.004	1.22 (1.06, 1.42)	0.006
13-17	1.74 (1.50, 2.03)	<0.001	1.73 (1.48, 2.02)	<0.001
Sex assigned at birth				
Male	Reference		-	
Female	0.95 (0.87, 1.04)	0.23	-	-
Number of other household members				
1	Reference		-	
2	1.38 (1.00, 1.91)	0.05	-	-
3-5	1.34 (0.99, 1.83)	0.06	-	-
>5	1.16 (0.83, 1.63)	0.39	-	-
Presence of chronic condition				
No	Reference		-	
Yes	0.98 (0.62, 1.54)	0.93	-	-
Perform physical activity regularly (≥3 days/week of physical activity of at least 30 minutes/day in the past six months)				
No	Reference		Reference	
Yes	0.84 (0.76, 0.93)	0.001	0.80 (0.72, 0.89)	<0.001
Self-reported SARS-CoV-2 infection before 7 December 2022				
No	Reference		-	
Yes	N.A.	N.A.	-	-
**COVID-19 vaccination status**				
Number of doses of COVID-19 vaccine received by the children (interval between the				

second dose of COVID-19 vaccine and 7 December 2022)				
2 doses (<1 month)	Reference		Reference	
2 doses (1-3 months)	3.33 (1.28, 8.50)	0.01	3.34 (1.29, 8.62)	0.01
2 doses (4-6 months)	4.93 (1.95, 12.49)	0.001	4.80 (1.89, 12.17)	0.001
2 doses (7-9 months)	6.28 (2.48, 15.90)	<0.001	6.17 (2.44, 15.66)	<0.001
2 doses (10-12 months)	5.82 (2.29, 14.77)	<0.001	5.90 (2.32, 15.00)	<0.001
2 doses (>12 months)	8.11 (3.21, 20.46)	<0.001	7.97 (3.15, 20.15)	<0.001

OR: crude odds ratios
AOR: adjusted odds ratios, odds ratios obtained from multivariate logistic regression models using all significant factors in the univariate analysis as candidates.
CI: confidence interval
